# Design of a Novel Wearable System for Foot Clearance Estimation

**DOI:** 10.3390/s21237891

**Published:** 2021-11-26

**Authors:** Shilpa Jacob, Geoff Fernie, Atena Roshan Fekr

**Affiliations:** 1 The Kite Research Institute, Toronto Rehabilitation Institute—University Health Network, Toronto, ON M5G 2A2, Canada; geoff.fernie@uhn.ca (G.F.); atena.roshanfekr@uhn.ca (A.R.F.); 2 Institute of Biomedical Engineering, University of Toronto, Toronto, ON M5S 3G9, Canada; 3 Department of Surgery, University of Toronto, Toronto, ON M5T 1P5, Canada

**Keywords:** fall prevention, tripping, foot clearance, gait analysis, wearable, time-of-flight sensor

## Abstract

Trip-related falls are one of the major causes of injury among seniors in Canada and can be attributable to an inadequate Minimum Toe Clearance (MTC). Currently, motion capture systems are the gold standard for measuring MTC; however, they are expensive and have a restricted operating area. In this paper, a novel wearable system is proposed that can estimate different foot clearance parameters accurately using only two Time-of-Flight (ToF) sensors located at the toe and heel of the shoe. A small-scale preliminary study was conducted to investigate the feasibility of foot clearance estimation using the proposed wearable system. We recruited ten young, healthy females to walk at three self-selected speeds (normal, slow, and fast) while wearing the system. Our data analysis showed an average correlation coefficient of 0.94, 0.94, 0.92 for the normal, slow, and fast speed, respectively, when comparing the ToF signals with motion capture. The ANOVA analysis confirmed these results further by revealing no statistically significant differences between the ToF signals and motion capture data for most of the gait parameters after applying the newly proposed foot angle and offset compensation. In addition, the proposed system can measure the MTC with an average Mean Error (ME) of −0.08 ± 3.69 mm, −0.12 ± 4.25 mm, and −0.10 ± 6.57 mm for normal, slow, and fast walking speeds, respectively. The proposed affordable wearable system has the potential to perform real-time MTC estimation and contribute to future work focused on minimizing tripping risks.

## 1. Introduction

Falls are a serious health concern for seniors in Canada as they are the primary cause of injury-related deaths, hospitalizations, and emergency department visits [1]. A study by Parachute reported that falls are the major contributor to overall injury costs in Canada, where falls “on the same level” had injury costs of 1.7 billion CAD in 2018 [1]. In addition to physical ramifications, falls can have a lasting negative impact on the quality of life of seniors and result in serious mental consequences such as fear of falling, loss of independence, and depression [2]. According to a study conducted by Blake et al., 53% of seniors’ falls are due to tripping [3]. This type of fall is typically the result of an unsuccessful or low Minimum Toe Clearance (MTC) that leads to unanticipated contact with the ground [4]. The MTC is the smallest distance between the toe and ground during the mid-swing phase of the gait cycle when the foot is at its maximum velocity [4]. The combination of high forward velocity, low MTC, and single-foot support can increase the risk of tripping and ultimately falls, especially for individuals with shuffling gait or limited lower limb movement [4,5]. Since a low MTC has been found to predict tripping risk, it is essential to develop a system that can accurately measure the MTC in real-time to minimize the likelihood of trip-related falls [6].

Many wearable systems currently exist that measure the MTC and other gait parameters. Inertial Measurement Units (IMUs) and infrared distance sensors are the most commonly used technologies to measure gait parameters, but each method presents its own challenges and limitations. There is still a need for a wearable system that can measure gait parameters with high accuracy using minimal hardware to be effectively incorporated into real-life applications.

In this paper, we propose a novel wearable system that can estimate the MTC accurately using two Time-of-Flight (ToF) sensors located at the toe and heel regions of the shoe. This affordable system was validated versus the motion capture (mocap) system, which is considered the ground truth. We discuss how to improve the measurements from the ToF signals by compensating the foot angle at each phase of the gait cycle and removing additional offsets in the ToF data.

## 2. Literature Review

Several methods have been used to measure the Minimum Foot Clearance (MFC) in an accurate, reliable, and unrestrictive manner. The most common method is the motion capture system, which uses multiple cameras positioned within an operating space to record three-dimensional movements of individuals based on the location of reflective markers [7]. Motion capture is considered the gold standard for estimating foot clearance due to its ability to provide precise and highly accurate measurements [7]. However, this system has a few drawbacks when used for gait analysis applications. For example, it requires a large amount of equipment, including several video cameras, computers to run the software, calibration wands, and reflective markers, making it expensive to operate and difficult to implement in settings outside the laboratory [8]. It also has a restrictive laboratory environment that compromises the natural mobility of participants that are aging or have gait impairments [7]. As a result, conducting gait analysis experiments with motion capture may not truly represent the real-life activities of these populations [7].

Wearable systems have been introduced as an inexpensive, portable, and lightweight alternative for gait analysis [7]. These systems provide flexibility when conducting research in outdoor and indoor environments without relying on large amounts of equipment, and minimize interference with an individual’s natural gait during everyday activities [7]. In the following sections, we summarized different wearable systems that use various technologies to measure gait parameters.

### 2.1. Inertial Measurement Units

IMUs are widely used to measure foot clearance parameters using a combination of accelerometer and gyroscope data [8]. The accelerometer measures the acceleration of the foot, and the gyroscope provides information about the angular velocity [8]. Previous papers have investigated the accuracy of foot clearance estimation using different configurations of the IMU in regards to where it is placed on the shoe (toe, heel, and instep) and the number of IMUs used [7,9]. In [10], Mariani et al. proposed a wireless foot-worn inertial sensor system to estimate the heel and toe clearance. The system consisted of one IMU located at the instep of the foot and measured 3-D acceleration and 3-D angular velocity [10]. After comparing the results to the motion capture system, it was found that the system had a Mean Error (ME) of 41 ± 23 mm for maximum heel clearance (MHC) and 13 ± 9 mm for MTC [10]. In [11], Kanzler et al. proposed a similar system to estimate toe and heel clearance using a single IMU, but the sensor was located on the lateral ankle. Results showed that the overall Mean Absolute Error (MAE) for heel clearance and toe clearance was 32.2 ± 15.0 mm and 16.9 ± 7.0 mm, respectively [11]. Benoussaad et al. also proposed a system with a single IMU placed at the ankle joint, which achieved a Root Mean Square Error (RMSE) of 7.4 mm for normal walking [8]. Kitagawa and Ogihara proposed a system that attached an IMU to the instep of the foot to measure acceleration and angular velocity [9]. The ME was about 2 ± 7 mm for foot clearance [9]. In a recent study, Fan et al. proposed a system that utilized a two-IMU configuration located near the toe and heel [7]. The study compared different configurations of IMUs to determine which design would provide the best estimate of the foot clearance when validated versus the motion capture system [7]. The two-IMU configuration had the best accuracy with an RMSE of 4.2 mm and 2.6 mm, and a ME of −3.4 ± 2.4 mm and 0.2 ± 2.6 mm for MHC and MTC, respectively [7]. This method provided the best accuracy due to their consideration of orientation estimation, sensor placement, and shock absorption [7].

Relying exclusively on IMU data to determine the foot displacement during walking provides different challenges in data analysis [7]. Angular velocity needs to be integrated to estimate the foot orientation, and a double integration needs to be performed on acceleration data to estimate the foot clearance [7]. The sensor noise also becomes integrated during these calculations, which introduces a significant amount of noise and drift over time [7]. Since foot clearance values lie within a small range of a few millimeters, the accumulating noise will affect the system’s accuracy [7]. The zero velocity updating technique has been used to reduce these estimation errors and correct the drift by resetting the velocity to zero when the foot is in contact with the ground [8]. However, this technique is only valid if the zero velocity intervals are detected accurately and reliably [12]. Due to the limited accuracy and indirect nature of measuring the distance with IMU-based systems, researchers have suggested adding more hardware such as pressure sensors or shock absorbers to the shoes, but this adds more weight and makes the shoes bulkier. Therefore, other sensors have been investigated to achieve high accuracy with fewer sensors and hardware [7].

### 2.2. Infrared Distance Sensors

Infrared (IR) distance sensors have become another option to measure foot clearance [13]. IR sensors are attached to the side of the shoe and calculate the distance based on the size of the reflected IR beam’s reception angle from the ground to the IR detectors on the shoe [13]. These sensors provide a direct method to measure the foot clearance without performing intermediate calculations that can introduce errors [7]. In [14], Kerr et al. proposed a system that used an optical proximity sensor located at the first metatarsal to measure foot clearance. The system demonstrated excellent agreement compared to the motion capture system with intraclass correlation coefficients of 0.93 for all speeds, 0.93 for normal speed, 0.97 for slow speed, and 0.89 for fast speed [14]. It is worth noting that these correlation values are estimated based on the signals for each step and not for the whole trial. In [13], Arami et al. had a two-IR configuration located near the toe and heel with an RMSE of 7.6 mm and 6.3 mm, and a ME of 0.8 ± 7.5 mm and 0.2 ± 6.3 mm for MHC and MTC, respectively. Tiwari and Joshi proposed a system that used two IR distance sensors at the toe and heel to detect gait events such as the heel-strike and toe-off, but did not use the system to estimate the foot clearance directly [15]. Although IR sensors reduce the error in vertical distance measurements due to their direct nature compared to IMU-based systems, they present limitations when considering the location of the sensor on the shoe, the range of the sensor, and the type of floor walked on [7]. Ground contours on different floor materials can introduce errors and affect the accuracy of IR sensors [7].

### 2.3. Ultrasonic Distance Sensors

Ultrasonic distance sensors were also investigated to measure the distance between the foot and ground through the transmission and reflection of ultrasonic waves [16]. In [16], Wahab et al. proposed a system that used an ultrasonic sensor and IMU at the heel to detect the foot angle and calculate the accurate MFC value [16]. The study found that determining the MFC using only distance data was insufficient and recommended incorporating acceleration data to provide a better real-time MFC estimation [16]. Qi et al. also proposed a system that attached an ultrasonic transmitter to the heel facing the direction of four receivers located at fixed positions [17]. Compared to motion capture, this system had an RMSE of 7.5 mm and an accuracy of 0.62 mm ± 7.48 mm for foot clearance [17]. Since ultrasonic sensors are based on the speed of sound, they can be sensitive to external factors such as the surrounding temperature, humidity, and nearby obstacles [17].

### 2.4. Pressure Sensors

Pressure sensors have also been used in addition to IMUs or distance sensors to detect gait events such as the heel-strike, toe-off, and foot flat moments [15]. They are often fitted into the foot insole in an unobtrusive manner to assist with zero velocity updating and increase estimation accuracy [15]. However, these sensors do not help quantify pressure values and are susceptible to wear and tear when worn over long periods [15]. Table 1 compares the error values for MTC, first maximum toe clearance (MX1), second maximum toe clearance (MX2), and MHC from wearable technologies described in the previous sections.

To overcome the limitations mentioned in this paper, we used ToF sensors with novel algorithms to provide accurate measurements of the foot clearance in different moments of the gait cycle. We achieved this without adding additional hardware to detect the gait phases or remove noise associated with different walking patterns.

## 3. Materials and Methods

### 3.1. System Design

**Figure 1:**
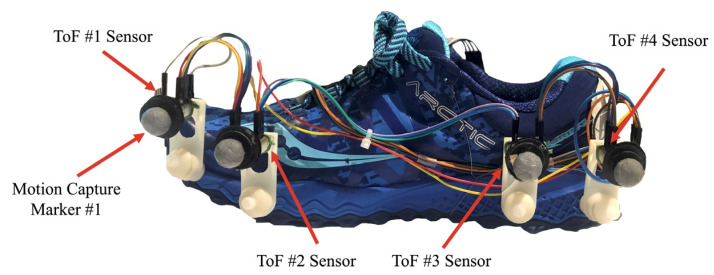
Design components of the proposed wearable system.

The new wearable system comprises four ToF sensors (SensorDots, Victoria, Australia) mounted on a running shoe, as shown in Figure 1. Since we want to evaluate the proposed system versus mocap as our reference, reflective markers are placed on the shoe at the same locations as each ToF sensor.

The ToF sensors use the VL53L0X laser time-of-flight sensor to measure distances ranging from 30 mm to 1200 mm with a 25° field of view. The sensor works by emitting an infrared light into the environment, which reflects on surrounding objects and returns to the sensor. The distance is measured by calculating the time it takes for the light to leave the device, bounce off nearby objects, and return to the device [18]. Since the ToF sensors have a minimum range where any distance measured below 30 mm will not be accurate [19], the sensors are placed on the shoe at the height of at least 50 mm to ensure that the measurements are reliable even when the foot reaches its minimum vertical distance. Custom 3D-printed mounts are used to secure each ToF sensor along the lateral edge of the shoe. The sensors communicate with the Metro M4 Express microcontroller (Adafruit Industries, New York, NY, USA), and the data is stored on an SD card data logging shield (Adafruit Industries, New York, NY, USA) at a sampling rate of 50 Hz. Six 1.5 V batteries are used to power the system and are placed in a box attached to the user’s waist using a waist bag. A 2.5 m ribbon cable is used to connect the components in the box to the sensors on the shoe.

### 3.2. Experiment Setup

**Figure 2:**
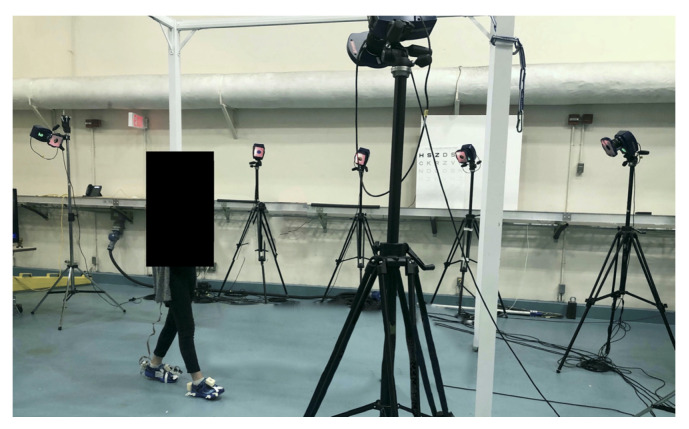
Experiment setup with the participant wearing the system and performing the walking task.

The experiment took place at the Challenging Environment Assessment Laboratory (CEAL) at the KITE Research Institute-Toronto Rehab (TRI)–University Health Network (UHN). A motion capture system with ten cameras (Motion Analysis, Rohnert Park, CA, USA) served as the ground truth for measuring the foot clearance. We recruited ten female adults (age 25.5 ± 4.1 years (mean ± SD), height 160.8 ± 4.2 cm, weight 56 ± 5.9 kg) without gait disorders to participate in this study. The study protocol was approved by the University Health Network Research Ethics Board (REB) and each participant provided consent prior to the study. During the experiment, participants were asked to walk on level ground at a self-selected normal (1.0 ± 0.2 m/s), slow (0.6 ± 0.1 m/s), and fast (1.4 ± 0.2 m/s) speed. Each participant completed two walking trials for each speed resulting in a total of 296 steps which consisted of 97 steps for normal speed, 115 steps for slow, and 84 steps for fast speed. All data was collected from the participant’s left foot. Figure 2 shows the setup with the participant wearing the wearable system and performing the experiment task.

## 4. Data Analysis

**Figure 3:**
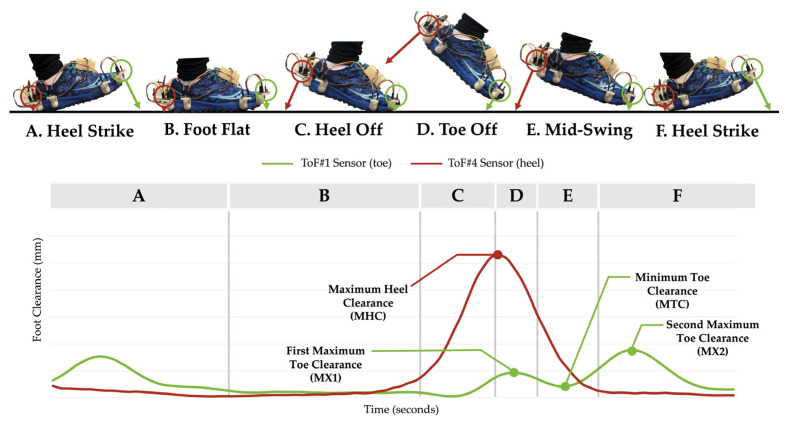
An example of a typical foot clearance graph with the key foot clearance parameters measured by the two ToF sensors located at the toe and heel. The arrows pointing from each ToF sensor represent the foot clearance measured at each phase of the gait cycle. Sections A to F represent each phase of the gait cycle.

In a typical foot-ground clearance graph, MTC, MX1, MX2, and MHC are identified as key foot clearance parameters [7]. These foot clearance parameters were extracted from the ToF signals located at the toe and heel areas, as shown in Figure 3.

First, the ToF sensors were calibrated to map raw sensor readings into corrected values and compensate possible systematic offset and gain [20]. We collected data at four predetermined heights, including 50 mm, 75 mm, 100 mm, and 125 mm, and used the Least-Square (LS) method to obtain the offset and gain values for each ToF sensor. These calibration parameters were applied anytime we collected data from the ToF sensors.

A low-pass filter with a passband frequency of 3 Hz and a stopband frequency of 6 Hz was applied to the mocap and ToF sensor data. To match the sampling rate of the ToF signal, the mocap signal was downsampled to 50 Hz. Since the data from both systems was not collected at the same time, the mocap and sensor signals were synchronized by finding and compensating the time lag between the two signals.

**Figure 4:**
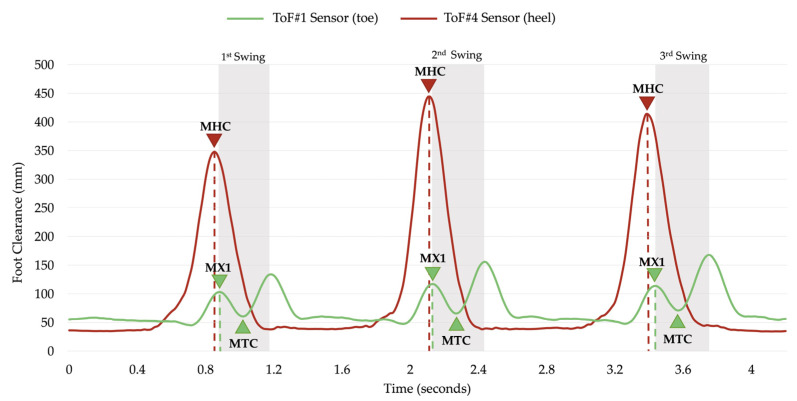
A sample of the distance signals collected from ToF #1 sensor and ToF #4 sensor for a 3-step trial captured from one participant during normal walking speed. The grey shaded area represents the swing phase of the gait cycle.

We developed a new algorithm to detect the MTC points in each stride. This is based on the fact that the MHC always occurs right before the MX1 peak since the heel-off phase occurs before the toe-off phase in the gait cycle (see Figure 4). The algorithm finds the time that the MHC occurs in the ToF #4 signal and starts searching for MX1 and MTC in ToF #1′s signal.

The lowest value in this region is detected as the MTC value for that gait cycle. This process is repeated for all consecutive gait cycles. The MTC detection algorithm was tested on the normal, slow, and fast walking speed data collected from the ToFs. The proposed algorithm was able to identify all the MTC moments for all speeds with 100% accuracy.

### 4.1. Foot Angle Compensation

**Figure 5:**
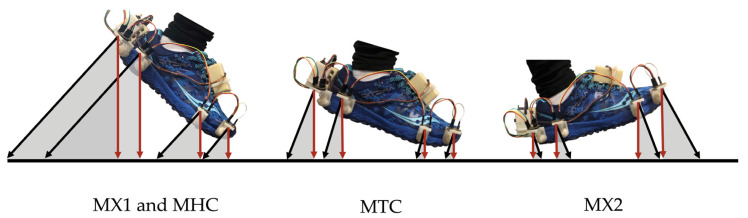
The foot angle at the different phases of the gait cycle. The black arrows represent the distance measured by the ToF sensors. The red arrows represent the actual foot-to-ground distance. The grey shaded regions represent the foot angle (β).

The ToF sensors measure the average distance of all objects within a 25° field of view [18]. The measured distance is approximately perpendicular to the sensor, as illustrated by the black arrows in Figure 5. When the foot moves through the different phases of the gait cycle, the sensor orientation is constantly changing, and as a result, the true foot-to-ground distance, shown by the red arrows in Figure 5, is not measured by the ToF sensors. The true foot-to-ground distance was determined by compensating the foot angle throughout each phase of the gait cycle.

**Figure 6:**
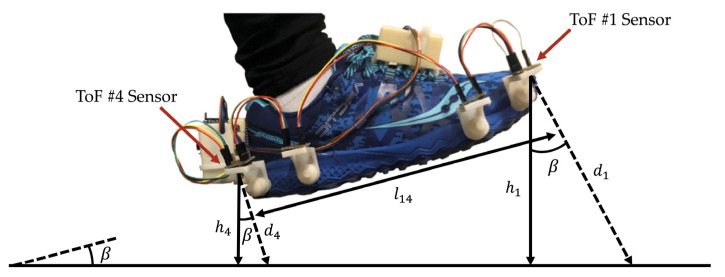
A labeled diagram of the foot angle, distance measurements, and sensor height to illustrate how the variables for Equations (1) and (2) were obtained to calculate the true foot-to-ground distance from each ToF sensor. This method was suggested by Arami et al. [13].

The foot angle was calculated using the distance measurements from the ToFs (see Figure 6) with the equation suggested by Arami et al. [13], presented below:

(1) $$Foot\;Angle=\beta =tan^{-1}\frac{d_{1}-d_{4}}{l_{14}},$$

where $l_{14}$ is the length measured between ToF #1 and ToF #4, and $d_{1}$ and $d_{4}$ are the distance measured by ToF #1 and ToF #4, respectively. Using the calculated foot angle, the height of the sensor ($h_{i}$), which is also the true foot clearance value, was computed using the following equation:

(2) $$h_{i}=d_{i}\times cos\beta ,$$

where $d_{i}$ is the distance measured from the ith ToF sensor, and β is the foot angle calculated in Equation (1).

### 4.2. Offset Compensation

**Figure 7:**
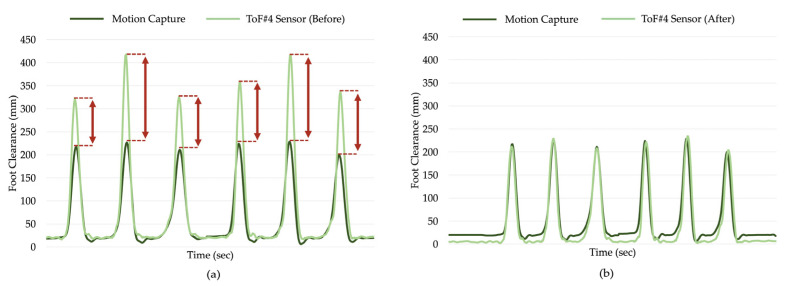
A sample plot of a 6-step normal walking trial of mocap and ToF #4 signals (**a**) before angle and offset compensation and (**b**) after angle and offset compensation. The red arrows in Figure 7a illustrate the difference in peak values measured by both systems.

Considering the signals from both the ToFs and mocap for all participants, we observed that the ToF signals always provided sharper peak values than the mocap data, especially at large distance measurements, as shown in Figure 7a. The study in [19] reported that the error systematically increases by distance for this type of ToF sensor, which might be the reason for the higher offset values observed in our experiments for MHC and MX1 compared to MX2 and MTC. After extracting the gait parameters, the offset was removed by randomly selecting 70% of the outputs from the mocap and ToF as the training dataset and calculating the difference between the two outputs to find the average offset error. The total average offset error was calculated separately for each ToF sensor at the normal, slow, and fast walking speed for each gait parameter. This step was randomly repeated five times, and the total average offset error (a constant value) was subtracted from the remaining 30% of the output data. Figure 7b shows an example of the foot clearance values from ToF #4 and mocap after angle and offset compensation.

Table 2 summarizes the compensated offset values for each gait parameter. We observed that the offset values were significantly different among various walking speeds. For example, considering ToF #4 for measuring MHC, the offset value for normal speed was about 14.68, and for fast speed, it was increased to 40.04. As shown in Table 2, the offset values for normal speed were less than the slow and fast speeds in most cases.

Another observation was that offset values were significantly different among each participant. To keep the system simple and general, we only removed the average offset values for each gait parameter, walking speed, and ToF sensor in this paper. In the future, we will customize this value based on each participant’s gait pattern. In a real-time application, the offset will be chosen automatically based on the walking speed for each participant to achieve more accurate ToF readings.

## 5. Results and Discussion

### 5.1. Correlation Coefficient

The Pearson’s correlation coefficient values for each speed and ToF sensor are summarized in Table 3. The values listed for each sensor are the average of the two trials completed for each walking speed. The average correlation coefficients for normal, slow, and fast walking speeds are 0.94 ± 0.06, 0.94 ± 0.05, and 0.92 ± 0.08, respectively. The overall correlation coefficient considering all ten participants, four ToF sensors, and all walking speeds is 0.93 ± 0.06.

It is important to note that the mocap and ToF signals captured from the heel (ToF #3 and ToF #4) were in greater agreement with an average *r* = 0.99 compared to the ToF sensors located on the toe (ToF #1 and ToF #2) with an average *r* = 0.88. This could be a result of the increased variation in the trajectory of the toe compared to the heel during walking. The correlation coefficient also significantly improved after compensating the foot angle at each phase of the gait cycle. Note that the correlation values were obtained by considering signals from each individual step separately. This means we did not include the stance phase of the gait cycle, where the correlation values might improve due to the constant distance values.

### 5.2. Foot Clearance Validation

**Figure 8:**
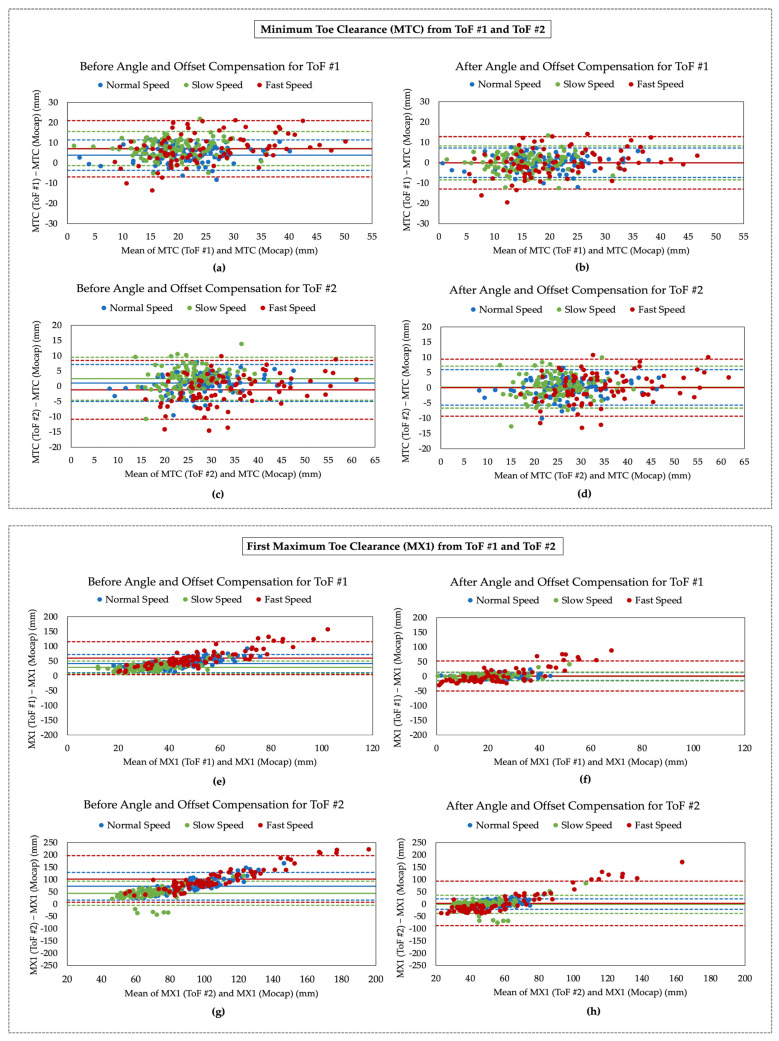

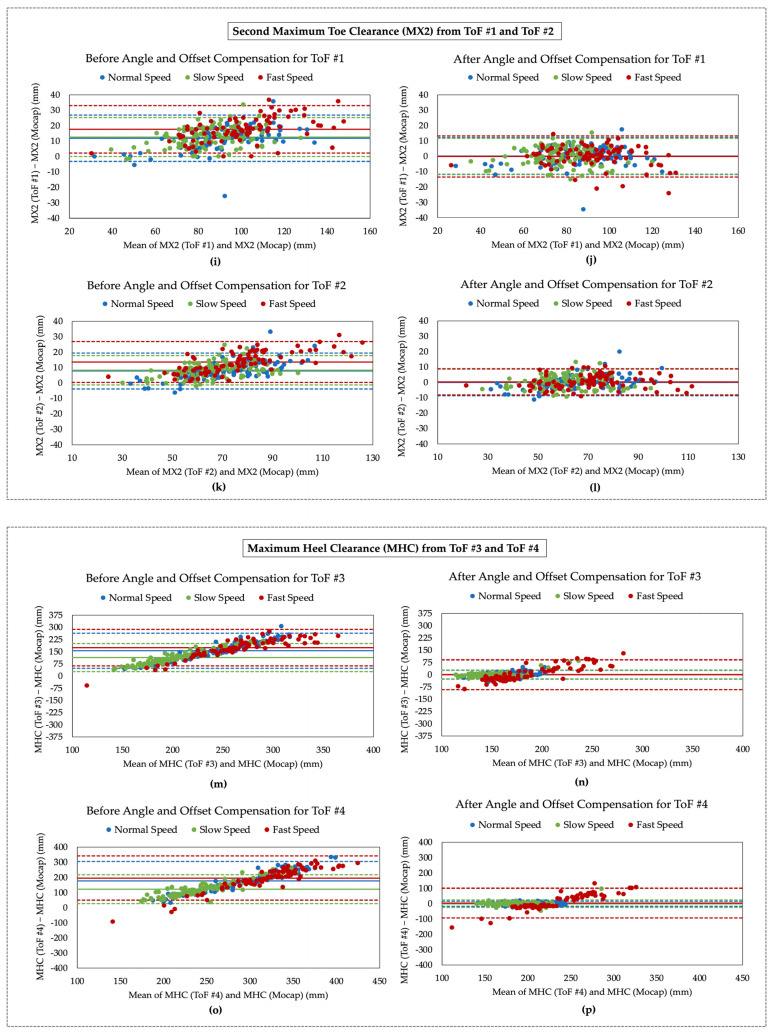
Bland-Altman plots for estimated gait parameters before and after angle and offset compensation for (**a**–**d**) Minimum Toe Clearance (MTC), (**e**–**h**) First Maximum Toe Clearance (MX1), (**i**–**l**) Second Maximum Toe Clearance (MX2) and (**m**–**p**) Maximum Heel Clearance (MHC) for normal, slow, and fast walking speed. The solid lines and dash lines represent the bias and limits of agreement (LOA) (Mean ± 1.96 SD), respectively.

To evaluate the accuracy of the proposed wearable system, Bland-Altman plots were generated to visualize the agreement between the mocap and ToF measurement systems. This plot quantifies the difference between measurements using a graphical method [21]. ToF #1 and ToF #2 were used to detect MTC, MX1, and MX2, while ToF #3 and ToF #4 were used to detect MHC. Figure 8 presents the Bland-Altman plots for each ToF sensor and gait parameter for normal, slow, and fast walking speeds before and after angle and offset compensation. A general observation for all plots was that the Limits of Agreements (LOAs) were significantly narrower after the angle and offset compensation. This means that applying these corrections to the ToF measurements resulted in a more precise agreement between the two systems. The average bias and the upper and lower LOAs for each gait parameter and walking speed are summarized in Table 4.

The positive bias values shown in Figure 8a,c,e,g,i,k,m, and o confirmed that the ToF sensors tended to overestimate the mocap measurements. These bias values were significantly improved (by 99.02% on average) after angle and offset compensation shown in Figure 8b,d,f,h,j,l,n,p. The bias values were close to zero for MTC and MX2, ranging from −0.12 mm to 0.26 mm. This suggests that the two measurement systems were producing similar results for MTC and MX2. The bias values for MX1 and MHC were larger, ranging from −0.95 mm to 4.47 mm.

There was also a positive trend observed for the MX1 and MHC plots before angle and offset compensation. These upward trends indicate that MX1 and MHC captured from the ToF sensors were overestimated in larger foot clearance values, whereas the estimations for smaller foot clearance were similar to ground truth. This is evident in Figure 7, where the ToF signals have noticeably larger peak values than the mocap signal, but the smaller distances are almost consistent between the two systems. It was also observed that this trend significantly decreased after angle and offset compensation shown in Figure 8f,h,n,p.

Figure 8 also illustrated that the slow speed (shown in green) typically had more consistent data points, which resulted in a smaller LOA region, whereas the fast speed (shown in red) had greater variability and, as a result, had a wider LOA. The ME and standard deviation, summarized in Table 5, were also calculated for each gait parameter and walking speed before and after compensation. The MEs decreased for all parameters after angle and offset compensation. The compensation did not improve the MTC values drastically since the foot does not make a large angle with the floor at this phase of the gait cycle (refer to Table 5). However, MX1, MX2 and MHC were significantly improved by the compensation due to the large foot angle at these gait phases. It was also observed that in most cases, the normal walking data had the lowest errors, while fast walking data had the highest errors.

For MTC, ToF #2 performed the best with an ME of 0.10 ± 2.98 mm for normal, 0.20 ± 3.52 mm for slow, and 0.02 ± 4.78 mm for fast walking speeds. For MX2, again ToF #2 provided the best results for all walking speeds. For MX1, ToF #1 performed best with an ME of −0.11 ± 6.92 mm, −0.20 ± 7.62 mm, and 1.11 ± 26.12 mm for normal, slow, and fast walking, respectively. ToF #4 had the lowest errors for MHC, considering all three walking speeds.

The majority of MTC values measured by ToF #1 ranged from 10 to 25 mm for the normal and slow walking speeds with all participants. However, the MTC values for the fast speed ranged from 10 to 40 mm, suggesting that the MTC had more variation and a tendency to be higher when participants walked fast. Interestingly, the MTCs from ToF #1 had a larger bias and wider LOAs than ToF #2 (demonstrated in Figure 8 and Table 4). This could be a result of the variability associated with bending and straightening the shoe, which occurs more often at the toe location. ToF #2 is located slightly closer to the middle of the shoe, so it is placed at a more stable location resulting in a more consistent signal. Another reason for the larger bias of ToF #1 with respect to ToF #2 could be due to its larger distance measurement range. It was also observed that the errors and standard deviations for MX1 and MHC were higher than the other gait parameters, especially during the fast walking speed.

The two-way ANOVA was used to investigate if there is a statistically significant difference between the mocap and ToF system and if the walking speed had significantly affected the gait parameters. All statistical analyses were performed using the JMP Pro software (Statistical Discovery, SAS, Cary, NC, USA), and statistical significance was considered when *p* < 0.05. The results revealed no significant difference in the mean values (*p* > 0.05) with an exception of MX1 measured by ToF #1 (*p* < 0.0001) and ToF #2 (*p* = 0.0425). This indicates that the mocap and wearable system produced similar outputs for MTC, MX2, and MHC after angle and offset compensation. It was also found that walking speed significantly affected each gait parameter but did not have a statistically significant impact on the correlation coefficient for each ToF sensor. When comparing the error at each ToF location, it was found that ToF 2 had the lowest error for MTC and MX2, ToF 1 had the lowest error for MX1, and ToF 4 had the lowest error for MHC. The difference between ToF locations was not statistically significant for MTC, MX2, and MHC, but was statistically significant for MX1.

As presented in Table 6, our results had the lowest mean error compared to previous studies for foot clearance estimation after applying angle and offset compensation. However, the standard deviation for our estimation was higher for MX1 and MHC, which could be due to the large variability between strides for these parameters. Both studies in [7,9] used IMUs, which rely heavily on the zero velocity updating method to obtain accurate results. This extra processing time may provide risks in real-time applications where every millisecond matters. All the data collections performed in the different studies were conducted in a short period of time, which might be why these differences are not visible when comparing various technologies in foot clearance estimation. Generally, each study has its own study protocol and data collection environment with specific equipment, sets of subjects, and methods. Therefore, this makes it difficult to compare the results of different studies. In some papers, such as [13], the authors did not report the number of participants. Therefore, we can conclude that the proposed system provides the best result by analysing data from ten participants considering a minimum number of sensors, hardware, and data processing.

In summary, the results demonstrated a strong agreement between the outputs from the proposed wearable system and mocap as our ground truth. Once angle and offset compensation techniques were applied to the ToF values, the ME, biases, and width of the LOAs decreased significantly. In addition, the underlying trends in the data were reduced, suggesting that the two measurement systems are producing similar results. Statistical analysis confirmed these results further by revealing no statistically significant difference between the ToF and mocap systems for most of the gait parameters after angle and offset compensation. The study included four ToF sensors to evaluate the data from each sensor and investigate if sensor location plays an important role in estimating the foot clearance. Based on the results, it is evident that all four ToF sensors can estimate the foot clearance with reasonable accuracy, but only ToF #1 and ToF #4 are necessary to estimate the foot clearance parameters. Therefore, we recommend that the wearable system’s final design should only include ToF #1 and ToF #4, and that ToF #2 and ToF #3 can be removed.

## 6. Limitations and Future Work

Due to the restricted capture volume of the mocap system, the number of steps taken by each participant for each speed was limited. For future studies, we will conduct the experiments in a larger area so that more gait cycles can be recorded for a more comprehensive analysis. Another study limitation was asking participants to walk at three self-selected speeds. It was difficult to determine if each participant had walked at the actual speed for each level (normal, slow, fast), and whether our results were comparable with other studies that conducted trials at different speeds. Therefore, in the future, we will control the speeds by using a metronome to make the results more consistent for each participant at each walking speed. Data collection for this study was conducted only on a level ground surface. In future studies, we will conduct experiments that include walking on inclined ground surfaces and walking up/down the stairs to investigate how these walking surfaces might affect the ToF measurements.

Another limitation was including only young, healthy female participants in the study. The mounts that secured the ToF sensors were drilled into a specific pair of running shoes, restricting our participant recruitment to include only those who fit into the shoe size. We understand that collecting data from this specific population limits the generalizability of the study results; however, the preliminary results were promising and confirmed the system’s feasibility to estimate the foot clearance. Future studies with a larger sample size, different genders, and footwear will be conducted. These future studies will investigate the proposed system’s ability to estimate the foot clearance on populations that could become possible end users, such as older adults and individuals with gait impairments.

The preliminary analysis of this study showed that ToF #1 and ToF #4 are sufficient for capturing the foot clearance parameters with acceptable accuracy. Another future direction would be optimizing the wearable design to include only ToF #1 and ToF #4 so that the system can be mounted on different types of footwear with maximum stability and minimum interference to the users. The next version of the system will also be evaluated outside the laboratory environment with more participants and footwear.

In addition, we plan to add a biofeedback mechanism to the system so that it can be used for both rehabilitation, specifically gait training, and as a warning tool to predict the risk of tripping and falling in everyday usage. For these purposes, we will first use machine learning techniques to recognize the user’s activities and then predict the user’s foot clearance in their next steps to activate a warning alert in the form of either vibration or sound.

## 7. Conclusions

A new wearable system was proposed that used ToF sensors to measure four different gait parameters: MTC, MX1, MX2, and MHC. Angle and offset compensation techniques were used to correct the ToF measurements and improve the system’s accuracy. The proposed system was validated against the reference motion capture system. The results showed that both systems had an excellent agreement and provided similar results for all gait parameters and walking speeds. Based on the study findings, we recommend that the system’s final design only includes ToF #1 and ToF #4 to estimate the foot clearance. The proposed wearable system offers a practical, lightweight, affordable, and accurate solution to estimate the MTC in real-time. It can be used as a tool to warn users of any possible risk of tripping and falling in their future steps. Therefore, it has a high potential to be integrated with assistive wearable technologies to minimize the risk of tripping and ultimately fall-related injuries.

## Figures and Tables

**Table 1:** Summary of the previous literature for wearable systems.

Ref.	# of Sub.	Type of Wearable System	MTC Error (mm)		MX1 Error (mm)		MX2 Error (mm)		MHC Error (mm)	
			Mean ± SD	RMSE	Mean ± SD	RMSE	Mean ± SD	RMSE	Mean ± SD	RMSE
[10]	12	1 IMU (instep)	13.0 ± 9.0	-	21.0 ± 15.0	-	−24.0 ± 18.0	-	41.0 ± 23.0	-
[8]	10	1 IMU (lateral ankle)	-	7.4	-	-	-	-	-	-
[9]	10	1 IMU (instep)	−1.8 ± 1.4	-	−0.8 ± 4.1	-	1.0 ± 3.4	-	-	-
[7]	8	2 IMUs + 2 shock absorbers (toe and heel)	0.2 ± 2.6	2.6	−1.3 ± 2.4	2.7	−0.3 ± 3.5	3.5	−3.4 ± 2.4	4.2
[13]	-	2 IR/ToF sensors (toe and heel)	0.2 ± 6.3	6.3	-	-	-	-	0.8 ± 7.5	7.6
[17]	10	1 Ultrasonic transmitter (heel) + 4 receivers	0.6 ± 7.5	7.5	-	-	-	-	-	-

Note: ‘-’ indicates that the information was not available in the literature.

**Table 2:** A summary of the offset means for each gait parameter, ToF sensor, and walking speed.

Gait Parameter	ToF Sensor	Offset Compensated (mm)		
		Normal Speed	Slow Speed	Fast Speed
MTC	ToF 1	3.46	6.92	6.38
	ToF 2	0.35	1.96	−2.16
MX1	ToF 1	21.82	20.60	38.41
	ToF 2	29.09	24.54	57.91
MX2	ToF 1	6.73	7.67	7.73
	ToF 2	4.06	4.58	6.18
MHC	ToF 3	20.44	28.29	47.36
	ToF 4	14.68	21.19	40.04

**Table 3:** The correlation coefficients between the mocap and ToF signals for each ToF sensor and walking speed.

	Normal Speed				Slow Speed				Fast Speed			
Sub #	ToF 1	ToF 2	ToF 3	ToF 4	ToF 1	ToF 2	ToF 3	ToF 4	ToF 1	ToF 2	ToF 3	ToF 4
1	0.91	0.88	0.99	0.99	0.90	0.88	0.98	0.98	0.86	0.72	0.97	0.98
2	0.95	0.95	0.99	0.99	0.93	0.96	0.99	0.99	0.93	0.91	1.00	0.99
3	0.94	0.94	0.99	0.99	0.93	0.94	0.99	0.99	0.91	0.89	0.99	0.99
4	0.93	0.94	1.00	0.99	0.89	0.91	0.99	0.99	0.93	0.90	0.99	0.99
5	0.91	0.87	0.98	0.99	0.90	0.87	0.98	0.98	0.88	0.76	0.98	0.98
6	0.87	0.85	0.99	0.99	0.87	0.88	0.99	0.99	0.81	0.82	0.99	0.99
7	0.86	0.92	0.99	1.00	0.84	0.90	0.98	0.99	0.85	0.91	0.99	0.99
8	0.89	0.85	0.99	0.99	0.93	0.88	0.99	0.98	0.81	0.80	0.99	0.99
9	0.80	0.84	0.99	0.99	0.83	0.82	0.98	0.98	0.82	0.88	0.99	0.99
10	0.88	0.86	0.99	0.99	0.92	0.88	0.99	1.00	0.90	0.86	0.99	0.99
**Average**	**0.89**	**0.89**	**0.99**	**0.99**	**0.89**	**0.89**	**0.98**	**0.99**	**0.87**	**0.84**	**0.98**	**0.99**

**Table 4:** The Bland-Altman limits of agreement (LOA) and mean before and after angle and offset compensation.

Gait Parameter	ToF #	Walking Speed	Before Angle and Offset Compensation			After Angle and Offset Compensation			Improvement on the Mean (%)
			Upper LOA (mm)	Lower LOA (mm)	Mean (mm)	Upper LOA (mm)	Lower LOA (mm)	Mean (mm)	
MTC	1	Normal	11.43	−3.64	3.89	7.14	−7.30	−0.08	102.06
		Slow	15.58	−1.29	7.14	8.21	−8.45	−0.12	101.68
		Fast	20.93	−6.93	7.00	12.78	−12.98	−0.10	101.43
	2	Normal	7.12	−5.04	1.04	5.93	−5.74	0.10	90.38
		Slow	9.51	−4.63	2.44	7.10	−6.70	0.20	91.80
		Fast	8.45	−10.87	−1.21	9.40	−9.35	0.02	98.35
MX1	1	Normal	72.27	10.83	41.55	13.45	−13.68	−0.11	100.26
		Slow	50.19	7.97	29.08	14.73	−15.13	−0.20	100.69
		Fast	115.72	4.36	60.04	52.30	−50.09	1.11	98.15
	2	Normal	129.20	16.37	72.79	21.57	−21.01	0.28	99.62
		Slow	93.37	−5.22	44.07	36.22	−38.13	−0.95	102.16
		Fast	196.79	7.44	102.12	93.69	−87.39	3.15	96.92
MX2	1	Normal	26.84	−3.18	11.83	12.25	−11.91	0.17	98.56
		Slow	25.18	0.00	12.59	11.69	−11.68	0.00	100.00
		Fast	33.04	2.26	17.65	13.28	−13.45	−0.09	100.51
	2	Normal	19.40	−3.90	7.75	8.56	−8.76	−0.10	101.29
		Slow	17.75	−1.27	8.24	8.57	−8.13	0.22	97.33
		Fast	26.83	0.35	13.59	8.80	−8.28	0.26	98.09
MHC	3	Normal	264.34	47.99	156.17	26.84	−25.99	0.42	99.73
		Slow	200.13	27.19	113.66	28.56	−27.85	0.36	99.68
		Fast	287.09	62.97	175.03	91.24	−90.83	0.20	99.89
	4	Normal	305.22	49.24	177.23	15.82	−16.82	−0.50	100.28
		Slow	218.40	27.35	122.87	24.30	−24.11	0.09	99.93
		Fast	341.09	50.01	195.55	101.27	−92.33	4.47	97.71

**Table 5:** The ME results of the proposed wearable system before and after angle and offset compensation for each gait parameter, ToF sensor, and walking speed.

Gait Parameter	ToF #	Normal Speed		Slow Speed		Fast Speed	
		ME before Compensation (mm)	ME after Compensation (mm)	ME before Compensation (mm)	ME after Compensation (mm)	ME before Compensation (mm)	ME after Compensation (mm)
MTC	1	3.89 ± 3.84	−0.08 ± 3.69	7.14 ± 4.30	−0.12 ± 4.25	7.00 ± 7.11	−0.10 ± 6.57
	2	1.04 ± 3.10	0.10 ± 2.98	2.44 ± 3.61	0.20 ± 3.52	−1.21 ± 4.93	0.02 ± 4.78
MX1	1	41.55 ± 15.67	−0.11 ± 6.92	29.08 ± 10.77	−0.20 ± 7.62	60.04 ± 28.41	1.11 ± 26.12
	2	72.79 ± 28.78	0.28 ± 10.86	44.07 ± 25.15	−0.95 ± 18.97	102.12 ± 48.30	3.15 ± 46.19
MX2	1	11.83 ± 7.66	0.17 ± 6.16	12.59 ± 6.43	0.00 ± 5.96	17.65 ± 7.85	−0.09 ± 6.82
	2	7.75 ± 5.94	−0.10 ± 4.42	8.24 ± 4.85	0.22 ± 4.26	13.59 ± 6.76	0.26 ± 4.36
MHC	3	156.17 ± 55.19	0.42 ± 13.48	113.66 ± 44.12	0.36 ± 14.39	175.03 ± 57.17	0.20 ± 46.45
	4	177.23 ± 65.30	−0.50 ± 8.33	122.87 ± 48.74	0.09 ± 12.35	195.55 ± 74.26	4.47 ± 49.39

**Table 6:** The accuracy values of the proposed wearable system.

Walking Speed	MTC (mm)		MX1 (mm)		MX2 (mm)		MHC (mm)	
	Mean ± SD	RMSE	Mean ± SD	RMSE	Mean ± SD	RMSE	Mean ± SD	RMSE
Normal	−0.1 ± 3.7	3.7	−0.1 ± 7.0	7.0	0.2 ± 6.2	6.2	−0.5 ± 8.3	8.3
Slow	−0.1 ± 4.3	4.6	−0.2 ± 7.6	8.3	0.0 ± 6.0	6.5	0.1 ± 12.4	13.4
Fast	−0.1 ± 6.6	6.1	1.1 ± 26.1	24.3	−0.1 ± 6.8	6.3	4.47 ± 49.4	46.1
**Mean**	**−0.1 ± 4.8**	**4.8**	**0.3 ± 13.6**	**13.2**	**0.0 ± 6.3**	**6.3**	**1.4 ± 23.4**	**22.6**

## Data Availability

Data will be made available on request to the correspondent author’s email with appropriate justification.

## References

[1] Parachute (2021). The Cost of Injury in Canada.

[2] Berg R.L., Cassells J.S. (1992). Falls in Older Persons: Risk Factors and Prevention.

[3] Blake A.J., Morgan K., Bendall M.J., Dallosso H., Ebrahim S.B.J., Arie T.H.D., Fentem P.H., Bassey E.J. (1988). Falls by elderly people at home: Prevalence and associated factors. Age Ageing.

[4] Santhiranayagam B.K., Lai D.T.H., Sparrow W.A., Begg R.K. (2015). Minimum toe clearance events in divided attention treadmill walking in older and young adults: A cross-sectional study. J. Neuroeng. Rehabil..

[5] Alcock L., Galna B., Perkins R., Lord S., Rochester L. (2017). Step length determines minimum toe clearance in older adults and people with Parkinson’s disease. J. Biomech..

[6] Begg R.K., Tirosh O., Said C., Sparrow W.A., Esteinberg N., Levinger P., Galea M. (2014). Gait training with real-time augmented toe-ground clearance information decreases tripping risk in older adults and a person with chronic stroke. Front. Hum. Neurosci..

[7] Fan B., Li Q., Liu T. (2020). Accurate foot clearance estimation during level and uneven ground walking using inertial sensors. Meas. Sci. Technol..

[8] Benoussaad M., Sijobert B., Mombaur K., Coste C.A. (2016). Robust Foot Clearance Estimation Based on the Integration of Foot-Mounted IMU Acceleration Data. Sensors.

[9] Kitagawa N., Ogihara N. (2016). Estimation of foot trajectory during human walking by a wearable inertial measurement unit mounted to the foot. Gait Posture.

[10] Mariani B., Rochat S., Büla C., Aminian K. (2012). Heel and Toe Clearance Estimation for Gait Analysis Using Wireless Inertial Sensors. IEEE Trans. Biomed. Eng..

[11] Kanzler C.M., Barth J., Rampp A., Schlarb H., Rott F., Klucken J., Eskofier B.M. Inertial sensor based and shoe size independent gait analysis including heel and toe clearance estimation. Proceedings of the 2015 37th Annual International Conference of the IEEE Engineering in Medicine and Biology Society (EMBC).

[12] Park S.K., Suh Y.S. (2010). A Zero Velocity Detection Algorithm Using Inertial Sensors for Pedestrian Navigation Systems. Sensors.

[13] Arami A., Raymond N.S., Aminian K. (2017). An Accurate Wearable Foot Clearance Estimation System: Toward a Real-Time Measurement System. IEEE Sens. J..

[14] Kerr A., Rafferty D., Dall P., Smit P., Barrie P. (2010). Using an Optical Proximity Sensor to Measure Foot Clearance During Gait: Agreement With Motion Analysis. J. Med. Devices.

[15] Tiwari A., Joshi D. (2020). An Infrared Sensor-Based Instrumented Shoe for Gait Events Detection on Different Terrains and Transitions. IEEE Sens. J..

[16] Wahab Y., Bakar N.A., Mazalan M. (2014). Error Correction for Foot Clearance in Real-Time Measurement. J. Phys. Conf. Ser..

[17] Qi Y., Soh C.B., Gunawan E., Low K.-S. (2014). Ambulatory Measurement of Three-Dimensional Foot Displacement during Treadmill Walking Using Wearable Wireless Ultrasonic Sensor Network. IEEE J. Biomed. Health Inform..

[18] (2018). Introduction to Time-of-Flight Long Range Proximity and Distance Sensor System Design. www.ti.com.

[19] MappyDot Registers and Instruction Set|SensorDots. https://sensordots.org/mappydotreg.

[20] Fekr A.R., Evans G., Fernie G. (2018). Walkway Safety Evaluation and Hazards Investigation for Trips and Stumbles Prevention. Adv. Intell. Syst. Comput..

[21] Giavarina D. (2015). Understanding Bland Altman analysis. Biochem. Med..

